# The Metabolic Response to Stress and Infection in Critically Ill Children: The Opportunity of an Individualized Approach

**DOI:** 10.3390/nu9091032

**Published:** 2017-09-18

**Authors:** Valentina De Cosmi, Gregorio Paolo Milani, Alessandra Mazzocchi, Veronica D’Oria, Marco Silano, Edoardo Calderini, Carlo Agostoni

**Affiliations:** 1 Pediatric Unit, Fondazione IRCCS Ca’ Granda Ospedale Maggiore Policlinico, 20122 Milan, Italy; valentina.decosmi@unimi.it (V.D.C.); gregorio.milani@unimi.it (G.P.M.); 2 Branch of Medical Statistics, Biometry, and Epidemiology “G. A. Maccacaro”, Department of Clinical Sciences and Community Health, University of Milan, 20122 Milan, Italy; 3 Department of Clinical Sciences and Community Health, Università Degli Studi di Milano, 20122 Milan, Italy; alessandra.mazzocchi@unimi.it; 4 Pediatric Intermediate Care Unit, Fondazione IRCCS Ca’ Granda Ospedale Maggiore Policlinico, 20122 Milan, Italy; 5 Pediatric Intensive Care Unit, Department of Anesthesia and Intensive Care and Emergency, Fondazione IRCCS Ca’ Granda, Ospedale Maggiore Policlinico, 20122 Milan, Italy; veronica.doria.vd@gmail.com (V.D.); edoardo.calderini@policlinico.mi.it (E.C.); 6 Unit of Human Nutrition and Health, Department of Food Safety, Nutrition and Veterinary Public Health, Istituto Superiore di Sanità, 00161 Rome, Italy; marco.silano@iss.it

**Keywords:** nutrition, indirect calorimetry, glycemic control, acute phase, ketosis, intensive care

## Abstract

The metabolic response to stress and infection is closely related to the corresponding requirements of energy and nutrients. On a general level, the response is driven by a complex endocrine network and related to the nature and severity of the insult. On an individual level, the effects of nutritional interventions are highly variable and a possible source of complications. This narrative review aims to discuss the metabolic changes in critically-ill children and the potential of developing personalized nutritional interventions. Through a literature search strategy, we have investigated the importance of blood glucose levels, the nutritional aspects of the different phases of acute stress response, and the reliability of the available tools to assess the energy expenditure. The dynamics of metabolism during stressful events reveal the difficult balance between risk of hypo- or hyperglycemia and under- or overfeeding. Within this context, individualized and accurate measurement of energy expenditure may help in defining the metabolic needs of patients. Given the variability of the metabolic response in critical conditions, randomized clinical studies in ill children are needed to evaluate the effect of individualized nutritional intervention on health outcomes.

## 1. Introduction

Acute stress conditions, such as sepsis or severe infections, are the major causes of admission into pediatric intensive care units (PICUs) [1]**.** The metabolic response to these conditions has precise pathophysiological mechanisms, clinical consequences, and therapeutic implications. It is divided into three phases—the acute phase, the stable phase, and the recovery phase—all characterized by specific neuroendocrine, metabolic, and immunologic modifications [2]. From an evolutionary perspective, the pathophysiological mechanisms contribute to the maintenance of body homeostasis, switching nutritional compounds towards different functions and, at a further stage, may facilitate recovery [3,4]. For instance, the translocation of amino acids (AAs) from muscle to liver leads to energy production and facilitates the synthesis of acute-phase proteins [3,5]. On the other hand, the metabolic response to stress also includes catabolic processes that, in many circumstances, may increase physiological instability and resource wasting [6]. The over-activation of inflammatory pathways (e.g., ubiquitin—proteasome system) may cause large protein breakdown, ending with the consumption of muscle tissues [7,8,9]. Furthermore, prolonged metabolic imbalance may lead to the development of abnormal energy expenditure, mitochondrial failure, and multiple cellular dysfunctions up to organs damage and failure [10]. In addition, infection-induced nutritional deficiencies appear to diminish immune responsiveness and impair antimicrobial defensive mechanisms, thus making the host more susceptible to secondary or opportunistic infections [9]. Considering these metabolic alterations and the risk of hyper- or hypo-metabolism [10], nutritional interventions may be crucial to improving the response of the host [11]. The aim of this narrative review is to discuss in view of the novel insights: (1) the pros and cons of a tight glycemic control; (2) the nutritional problems and strategies in the different phases of acute stress response; and (3) the reliability and limitations of the available tools to assess energy expenditure.

## 2. Methods—Literature Search Strategy

Electronic databases (Pubmed, Medline, Embase, Google Scholar, Knowledge Finder) were used to locate and appraise relevant studies. We carried out the search to identify the articles published in English on (i) glycemic control, (ii) nutritional interventions in the different acute stress phases, (iii) the role of the parenteral nutrition and of the immunity-enhancers nutrients and, finally, (iv) the usefulness of predictive equations and indirect calorimetry use in critically ill children. Relevant articles published from January 2007 to March 2017 were identified using the following groups of key terms: “metabolic response” AND “critical illness” AND “infections”; “hyperglycemia” AND “critically ill children” OR “nutritional status” OR “nutrition” OR “nutritional sciences” AND “critical care”; OR “hospitalized children” OR “child, hospitalized” “insulin therapy” AND “critically ill children”; “energy expenditure” AND “critically ill children”; “nutrition” AND “critically ill children” OR “PICU”. RCT and largest non-RCT studies were considered. In the eligible studies, we focused on data on infective events (or inflammation status), need of mechanical ventilation, length of PICU stay, and mortality rate (number of deaths) for the different nutritional approaches.

## 3. Results

### 3.1. Hyperglycemia and Glycemic Control in Stress Conditions

Stress hyperglycemia remains an unsolved medical condition. It is usually defined as blood glucose level >11.1 mmol/L [12]. Its incidence is very high, ranging between 56% and 86% among patients requiring intensive care [12]. Mechanical ventilation, vasopressor/inotropic infusion, continuous renal replacement therapy, infections, and long lengths of stay are the main risk factors of hyperglycemia that are commonly associated with worse outcomes [1].

Several trials have investigated the consequences of glycemic alterations and of their correction, providing inconclusive results. The effect of targeting age-adjusted normoglycemia with insulin infusion was investigated in a large prospective, randomized, controlled study including 700 critically-ill pediatric patients. Subjects were randomly assigned to intensive insulin treatment (to target blood glucose concentrations of 2.8–4.4 mmol/L in infants and 3.9–5.6 mmol/L in children) or conventional insulin infusion to prevent blood glucose from exceeding 11.9 mmol/L. A significantly shorter PICU stay was found in the intensively treated group (5.5 days vs. 6.2 days, *p* = 0.017) [13]. However, among several neurocognitive outcomes analyzed in a four-year follow-up of these children, only motor coordination (*p ≤* 0.03) and cognitive flexibility (*p* = 0.02) were worse in conventional insulin infusion group [14]. Another study on 97 patients with a median age of two years demonstrated that hyperglycemia was associated with higher morbidity (e.g., need of mechanical ventilation at 30 days) in meningococcal sepsis [15]. Other investigators found significantly reduced number of complications (e.g., incidence of sepsis) with lower (6.7–7.2 mmol/L) compared to higher (8.3–8.9 mmol/L) glucose targets in pediatric patients with burns [16]. 

On the contrary, a large multicenter trial, including more than 1300 critically-ill children randomly assigned to conventional glycemic control (glucose below 12 mmol/L) or tight glycemic control (4–7 mmol/L), found that the tight glycemic control had no significant effect on need of mechanical ventilation and mortality [17]. In this study, however, the upper limit of tight glycemic control was higher than in previous RCT, reducing the number of patients undergoing insulin treatment. In “The Heart and Lung Failure–Pediatric Insulin Titration (HALF-PINT)” trial (Clinical Trials ID: NCT01565941), glycemic control targeted to blood levels between 2.8–4.4 mmol/L in infants and 3.3–5.6 mmol/L in children was neither associated with lower length of PICU stay nor with mortality, as compared to levels between 8.3 and 10 mmol/L [18].

It may also be considered that tight glycemic control is difficult to achieve and, very often, intensive insulin therapy inevitably increases the risk of hypoglycemic episodes [19]. In turn, if severe and prolonged, low glucose levels may also cause major adverse events. A study investigating the effects of hypoglycemia found that critically-ill infants spending more than 50% of the time with lower (4.4–6.1 mmol/L) glucose levels had more frequent complications (e.g., renal failure) than those with higher (>11.1 mml/L) glucose levels [20]. However, in the Leuven pediatric study, although hypoglycemia was more common in children on intensive insulin therapy and patients developing hypoglycemia had a higher risk of death, this association was not significant and it could be explained by duration of PICU stay [13,14]. Other similar studies showed contrasting results on the association between hypoglycemia episodes and neurological consequences [21,22].

Randomized clinical trials on glycemic control and nutrition in critically-ill children are reported in Table 1. As a result of this section, the targets of glycemic control are still debated. Since normoglycemia is mostly associated with favorable outcomes, in terms of hospital stay and mortality, both hyperglycemia and hypoglycemia should be adequately prevented or promptly managed. However, the adoption of a tight glucose range for critically-ill patients is debated [10]. Indeed, currently, there is no recommendation available on hyperglycemia management provided from pediatric international societies. A “common sense approach” suggests keeping blood glucose between 7.8 and 10.0 mmol/L [23].

### 3.2. The Acute Phase: Metabolic Steps and Nutritional Implications

In acute stress conditions, circulating glucose and glycogen stores are rapidly depleted. Hence, hepatic gluconeogenesis, fatty acid beta-oxidation, and ketogenesis become the primary source of energy. At a further stage, the energy necessary for the increased gluconeogenesis is provided from either lactate or proteins and AAs [24]. These physiologic mechanisms are mediated by the insulin release switch-off followed by a production of glucagon, cortisol, and epinephrine, and activation of the sympathetic activity. They aim at providing sufficient energy for body metabolism and, among them, ketone bodies mainly supply central nervous energy requirements. During severe infections increased levels of inflammatory cytokines (e.g., IL1 and TNF), ACTH, and growth hormone tend to amplify these pathways and enhance protein breakdown [25,26,27]. Although these mechanisms are well known, management of ketosis in acute stress conditions is still challenging. On the one hand, ketone bodies are organic acids and, therefore, consume bicarbonates leading to blood acidosis and may cause malaise, nausea, and vomiting [25,28]. On the other hand, the amount of fatty acids resulting from lipolysis may even exceed energy requirements and glucose supplementation may rapidly lead to hyperglycemia and hepatic steatosis [24,25]. Yet, no randomized control trial has been conducted so far on different strategies to manage ketosis in critically-ill children.

Failing to provide adequate amounts of nutrients in the acute phase of stress response also results in exacerbation of existing nutritional problems in children. Malnutrition and infection may indeed interact, reinforcing each other even in milder stage of disease [29,30,31]. Furthermore, restricted nutritional support may stimulate autophagy, a survival mechanism by which cells break down their own damaged components to recycle intracellular nutrients and generate energy during starvation [32,33,34]. However, the acute stress response is a complex condition where hypercatabolism and muscular tissue consumption often cannot be reversed even with increased provision of nutrients (“futile cycle of nutrients”) [35]. On the other hand, overfeeding, i.e., a caloric intake/resting energy expenditure (REE) ratio >110% or >120% [36], is associated with increased morbidity (e.g., delayed ventilator weaning), prolonged hospitalization, and a higher mortality [37]. Overfeeding may also inhibit autophagy, leading to an increased risk of cell death and organ dysfunction [35]. Being the lower and upper limits of individual energy requirements unknown in critically-ill children and may largely vary in the acute phase of stress conditions, a characteristic paradigm of under- and overfeeding is the unpredictable combination of metabolic and feeding patterns [38]. 

### 3.3. Stable and Recovery Phases

During the stable phase, both an early normalization of the catabolic counter-regulatory hormone levels and an increased effect of anabolic hormones occur, however, proteins continue to be wasted while fat stores remain relatively intact [39]. During this phase, the risk of muscle atrophy remains high, especially if this condition lasts several weeks. On the contrary, in the recovery phase, protein synthesis exceeds protein break down. Nutrition in both these phases should slowly increase to allow recovery and growth [40]. A recent systematic review and a single-center study in mechanically-ventilated children calculated a minimum intake of, respectively, 57 and 58 kcal/kg/day to achieve a positive nitrogen balance [41,42]. In both studies, a protein intake of 1.5 g/kg/day was suggested to attain nitrogen balance. In these studies, however, no difference was made between the stable and recovery phases.

### 3.4. Nutrition: Method of Administration and Immunity-Enhancers Nutrients

It is generally accepted that enteral nutrition is advised in the stable phase and mostly in the recovery phase. In the acute phase, it may also be of benefit, but its composition and timing of administration should be cautiously considered [43].

A large RCT, including 1440 patients from three different PICUs, found that early (within 24 h) parenteral supplementation of AAs was associated with a higher rate of infections and longer PICU stay [44]. Yet, the heterogeneity of the population and the different glycemic control strategies were likely to be biased across the participating centers [43,44]. On the contrary, in a retrospective study of more than 5100 critically-ill children, early enteral nutrition, over the first 48 h of admission, was associated with a lower mortality rate in those with a PICU length of stay at least 96 h [45].

Many strategies to optimize enteral nutrition have been developed recently. It has been suggested that an immune-enhancing formula (i.e., giving patients perioperative nutritional supplements with immunonutritional additives) might improve the general and metabolic conditions in adults with infection [5]. In particular, the importance of AAs, dietary nucleotides, and lipids in modulating immune function has been recognized. For instance, the arginine plasma concentrations are strongly related to the severity of systemic inflammation, being especially low during the acute phase of critical illness [46]. Dietary supplementation with arginine might have positive effects on immune function and reparative collagen synthesis [47]. The role of glutamine supplementation is controversial: experimental work proposed various mechanisms of action, but none of the randomized studies in early life showed any effect on mortality and only a few showed some effect on inflammatory response, organ function, and a trend for infection control [47,48]. Briassoulis et al., in a blinded, randomized, controlled trial, compared nitrogen balance, biochemical indices, antioxidant catalysts, and clinical outcomes in critically-ill children given an immune-enhancing formula or conventional early enteral nutrition [49]. In their cohort, immunonutrition had a favorable effect on some biochemical indices (e.g., natremia) and antioxidant catalysts. However, the mortality rate did not differ between the two groups [49]. A further single-center, randomized, blinded controlled trial in 38 children with septic shock, performed by the same group, compared the effect of early enteral feeding using immune-enhancing with non-immune-enhancing formulas on cytokines. The study showed that immune-enhancing nutrition can interfere with the production of interleukin-6, but no evidence was found regarding the impact on the short-term outcome [50]. Finally, no clinical effect was provided by the immune-enhancing diet in a study including 40 ventilated children with severe head injury [51].

Overall, the present section suggests that, besides the role of the appropriate timing of the parenteral and enteral nutrition, some specific nutrients, particularly AAs, may contribute to an improvement of the immune response. Newer formulations of enteral or parenteral mixtures of AAs meeting the individual needs of different critically-ill populations should be tested [10].

### 3.5. Energy Expenditure Assessment

In critically-ill children, measured or calculated REE has been proposed to estimate energy intake requirements in children [2]. The five most commonly-used REE prediction formulas are: the World Health Organization (WHO) formula, the Harris–Benedict formula, the Schofield formula based on weight, the Schofield formula based on weight and height, and the Oxford formula [25]. These formulas have been validated in healthy children. However, they were found inaccurate in critically-ill children [38,40,52]. In a prospective cohort study, performed in a PICU setting, standard equations overestimated the energy expenditure and an 83% incidence of overfeeding with cumulative energy excess of up to 8000 kcal/week was observed [11]. Another prospective study found that measured energy expenditure (MEE) during critical illness was much lower than the energy expenditure predicted by formulas [53]. Further studies came to similar conclusions [54,55]*.*

IC has been proposed as a reference method to measure REE. This technique is used to measure the rate of energy production and substrate oxidation in children, both in clinical and research settings. The recent clinical guidelines of the American Society for Parenteral and Enteral Nutrition (ASPEN) for nutritional support of the critically-ill child, suggest that IC measurements should be obtained in patients with suspected metabolic alterations or malnutrition [6]. A study including 150 patients found that 72% of PICU patients were candidates for IC accordingly to the ASPEN guidelines [56]. Particularly, authors suggested prioritizing performing IC in patients <2 years of age, malnourished (underweight/overweight) on admission, or with a PICU stay of >5 days [56].

In recent years, the reliability of ventilator-derived carbon dioxide production (VCO_2_) equations was investigated. These simplified methods measure the VCO_2_ derived from measurements of exhaled gas volume and carbon dioxide (CO_2_) concentrations and seem to be a promising tool when IC is not available or applicable [57]. Recent studies have demonstrated that this technique is a promising option for the determination of energy requirements in children on mechanical ventilation [30]. However, since the variability of RQ (respiratory quotient) influences the accuracy of the EEVCO_2_ calculation (EE from CO_2_ measurements), and many of these approaches assume that the RQ value is a fixed value*,* the validity of this technique as an alternative to IC is questionable in some circumstances [57].

Strides have been made to build new, compact metabolic monitors to measure REE in PICU and to validate them. However, again, there is a wide range of conditions that may compromise their accuracy. For instance, metabolic monitors’ errors were shown to be significantly affected by oxygen concentration and minute ventilation and when used during inhaled anesthesia [58].

## 4. Conclusions

For critically-ill children, the role of nutrition is evolving from a simple supportive function to the possibility of an effective co-adjuvant therapy. However, the variability of metabolic responses to stress requires testing the hypothesis of an individualized approach to nutrition in critically-ill children, since available data are mainly derived from healthy individuals [59]. Additionally, the possible “intermediary” role of the microbiome should be investigated in future studies in PICUs [59]. Unfortunately, as previously mentioned, in stress conditions, the futile cycle of nutrients may make most interventions that are effective in healthy subjects useless. Indeed, inconclusive data are available in most important issues of critically-ill child nutrition (such as the glycemic control) and no international recommendation exists for the management of many common problems in their day-to-day care [10]. The determination of individual macronutrients needed in the various phases of stress are still more hypothetical than evidence-based. Finally, problems with the current predictive equations and lack of availability of IC are likely to result in continued under- or overfeeding in many critically-ill children, with the associated morbidity [60]. New efforts are urgently needed to develop individualized nutrition strategies and evaluate their effect on relevant health outcomes.

## Figures and Tables

**Table 1:** Randomized clinical trials on glycemic control and nutrition in critically-ill children.

Authors, years	Country	Groups	Primary Outcomes	Results
Vlasselaers et al. [13]	Belgium	Tight glycemic control (interventional group) *n* = 700, conventional glycemic control (control group) *n* = 351, age = 0–16 years. Statistical power = 80%	Effect of tight glycemic control on duration of PICU stay and inflammation	PICU stay was shorter (5.5 vs. 6.2 days, *p* = 0.017) and C-reactive protein change after 5 days lower (–9.8 mg/L vs. 9.0 mg/L, *p* = 0.007) in interventional vs. control group
Mesotten et al. [14]	Belgium	Tight glycemic control (interventional group) *n* = 222, conventional glycemic control (control group) *n* = 234, age ≤ 16 years. Statistical power = 80%	Effect of tight glycemic control on long-term follow-up of neuro-cognitive-outcomes	No significant difference between the two groups was observed
Jeschke et al. [16]	USA	Tight glycemic control (interventional group) *n* = 60, conventional glycemic control (control group) *n* = 159, age = 0–16 years. The study was underpowered	Effect of tight glycemic control on infectious events	Sepsis was less frequent (*p* < 0.05) in interventional than in control group (8.2% and 22.6% of patients, respectively)
Macrae et al. [17]	England	Tight glycemic control (interventional group) *n* = 694, conventional glycemic control (control group) *n* = 675, age = 0–16 years. Statistical power = 80%	Effect of tight glycemic control on days alive and free from and free from mechanical ventilation at 30 days after enrollment	No significant difference between the two groups was observed
Agus et al. [18]	USA	Tight glycemic control (interventional group) *n* = 349, conventional glycemic control (control group) *n* = 360, age = 2 weeks–17 years. Statistical power = 80%	Effect of tight glycemic control on length of PICU stay	No significant difference between the two groups was observed
Agus et al. [19]	USA	Tight glycemic control (interventional group) *n* = 490, conventional glycemic control (control group) *n* = 490, age = 0–36 months. Statistical power = 80%	Effect of tight glycemic control on mortality, length of PICU stay, and infectious events	No significant difference between the two groups was observed
Sadhwani et al. [21]	USA	Tight glycemic control (interventional group) *n* = 121, conventional glycemic control (control group) *n* = 116, age = 0–36 months. Statistical power not reported	Effect of tight glycemic control on neurodevelopment follow-up	No significant difference between the two groups was observed
Vanhorebee et al. [22]	Belgium	Tight glycemic control (interventional group) *n* = 349, conventional glycemic control (control group) *n* = 351, age = 0–16 years. Statistical power = 80%	Effect of tight glycemic control on neurological injury biomarkers	No significant difference between the two groups was observed
Vanhorebee et al. [44]	Belgium, Netherlands, Canada	Early parenteral nutrition (interventional group) *n* = 723, late parenteral nutrition (control group) *n* = 717, age = 0–17 years. Statistical power = 70%	Effect of macronutrients supplementation timing on infections, need of mechanical ventilation, and length of PICU stay	The early provision of amino-acids, and not glucose or lipids, was associated with worse outcomes
Briassoulis et al. [49]	Greece	Immunonutrition (interventional group), *n* = 25. Conventional enteral nutrition (control group) *n* = 25, age = 8–9.2 years. Statistical power not reported	Effect of immunonutrition on biochemical nutritional markers and hard outcomes (mortality, length of PICU stay, and need of mechanical ventilation)	Immunonutrition had a favorable effect on few nutritional biochemical markers, but not on hard outcomes
Briassoulis et al. [50]	Greece	Immunonutrition (interventional group), *n* = 15, conventional enteral nutrition (control group) *n* = 15, age = 6.5–7.9 years. Statistical power not reported	Effect of immunonutrition on interleukins in septic children	IL-6 levels were lower (11.8 vs. 38.3 pg/mL, *p* < 0.001) and IL-8 higher (65.4 vs. 21 pg/mL, *p* < 0.03) in interventional group compared with control group
Briassoulis et al. [51]	Greece	Immunonutrition (interventional group), *n* = 20, conventional enteral nutrition (control group) *n* = 20, age = 6–10.5 years. Statistical power not reported	Effect of immunonutrition on biochemical nutritional markers and hard outcomes (mortality, length of PICU stay, and need of mechanical ventilation) in severe head injury patients	Except for IL-8 levels and nitrogen balance, no difference was observed between the two groups

PICU, pediatric intensive care unit.
